# Orofacial Pain Management: An Overview of the Potential Benefits of Palmitoylethanolamide and Other Natural Agents

**DOI:** 10.3390/pharmaceutics15041193

**Published:** 2023-04-09

**Authors:** Simona Santonocito, Martina Donzella, Pietro Venezia, Giada Nicolosi, Rodolfo Mauceri, Gaetano Isola

**Affiliations:** 1 Department of General Surgery and Surgical-Medical Specialties, School of Dentistry, University of Catania, 95124 Catania, Italy; 2 Department of Surgical, Oncological, and Oral Sciences, University of Palermo, 90127 Palermo, Italy

**Keywords:** inflammation, lipid mediator, molecular pathways, natural drugs, neuropathic mediators, nutraceutical agents, oral–facial pain, palmitoylethanolamide, periodontitis, teeth

## Abstract

Pain is the most common symptom that dentists are confronted with, whether acute (pulpitis, acute periodontitis, post-surgery, etc.) or chronic diseases, such as periodontitis, muscle pain, temporomandibular joint (TMJ) disorders, burning mouth syndrome (BMS), oral lichen planus (OLP) and others. The success of therapy depends on the reduction in and management of pain through specific drugs, hence the need to analyze new pain medications with specific activity, which are suitable for long-term use, with a low risk of side effects and interactions with other drugs, and capable of leading to a reduction in orofacial pain. Palmitoylethanolamide (PEA) is a bioactive lipid mediator, which is synthesized in all tissues of the body as a protective pro-homeostatic response to tissue damage and has aroused considerable interest in the dental field due to its anti-inflammatory, analgesic, antimicrobial, antipyretic, antiepileptic, immunomodulatory and neuroprotective activities. It has been observed that PEA could play a role in the management of the pain of orofacial origin, including BMS, OLP, periodontal disease, tongue a la carte and temporomandibular disorders (TMDs), as well as in the treatment of postoperative pain. However, actual clinical data on the use of PEA in the clinical management of patients with orofacial pain are still lacking. Therefore, the main objective of the present study is to provide an overview of orofacial pain in its many manifestations and an updated analysis of the molecular pain-relieving and anti-inflammatory properties of PEA to understand its beneficial effects in the management of patients with orofacial pain, both neuropathic and nociceptive in nature. The aim is also to direct research toward the testing and use of other natural agents that have already been shown to have anti-inflammatory, antioxidant and pain-relieving actions and could offer important support in the treatment of orofacial pain.

## 1. Introduction

Pain is an unpleasant sensory and emotional experience that may be associated with actual or potential tissue damage [1]. It is not always associated with a biological benefit or a synonym for warning against tissue damage or wound healing; sometimes, it is instead a nonprotective and unhealthy pain [2,3]. Orofacial pain has a prevalence of about 1.9 percent, is higher in the female sex and the diagnosis, evaluation and management of related disorders often involve several specialists, as it is a complex process [4]. Pain affecting the orofacial region can arise from the dentoalveolar tissues, be muscular in nature and come from the temporomandibular joint (TMJ), hence the importance of its proper identification and careful objective examination to distinguish its origin [5]. Very often, dental diseases are the protagonists of this soreness. The type of pain most frequently found in the oral–facial region is induced by the acute stimulation of nociceptors by stimuli of various kinds at the level of the oral cavity; this is called nociceptive pain [6]. The excitation of nociceptors results in different types of pain depending on the tissue involved (e.g., pulpal tissue or periodontal tissue) [7]. The main proponent of acute facial pain is generally represented by the trigeminal nerve via the mandibular and maxillary branches, resulting in deep somatic pain [8], but involvement or inflammation of other structures of the dental elements (e.g., pulpitis) can cause considerable pain and discomfort [9]. Very common diseases, such as oral lichen planus (OLP), are often the cause of oral mucosal lesions and protopathic orofacial pain [10], but nociceptive pain could also originate from the TMJ because of inflammation or degradation of the joint’s own structures [11].

Neuropathic pain, on the other hand, with an estimated prevalence in the population of around 1–1.5% [12], is determined by a primary or secondary lesion of the peripheral or central nervous system, conditions such as burning mouth syndrome (BMS), trigeminal neuralgia and post-herpetic neuralgia are not easy to diagnose, so even treatment sometimes fails to be resolving and effective [13,14]. These are conditions that greatly affect everyday life in sufferers, such as BMS, a syndrome characterized by persistent oral burning in the absence of any actual organic disorder to which the symptomatology can be traced [15]. Although still the subject of studies to understand its origin and etiology, only hypotheses have been made about it; generally, the diagnosis is differential to exclude other diseases [16], and recently, evidence of central nervous system involvement with altered dopaminergic control has emerged [17,18].

One of the most widely used approaches for orofacial pain is the mechanistic approach, which involves intraoral devices, such as splints, braces and prostheses, in order to achieve a neuromuscular-guided ideal position or a condylar-guided ideal position capable of having the pain reduced. Very often, such an approach is carried out without the right clinical insights into the origin and pathogenesis of the treated pain and without having made the right differential diagnosis, leading to treatment failure [19,20].

Therefore, reducing the management of orofacial disorders to an exclusive mechanistic approach is reductive as the pathogenesis of these disorders may require different or even combined approaches (mechanistic and pharmacological). Finally, it is important to consider suffering patients as complex and unique individuals who may express and manifest pain in very different ways and who, therefore, require different and individualized approaches [21].

The therapies implemented to date aim to enable those with orofacial pain, whether nociceptive or neuropathic, to resolve or reduce the related symptomatology without excessive side effects. In this regard, natural agents that have a targeted anti-inflammatory and pain-relieving action have long been studied that may be appropriate in resolving the problem. Palmitoylethanolamide (PEA), or a bioactive lipid mediator discovered by Rita Levi-Montalcini, has been explored in this regard [22,23]. It is synthesized in all tissues of the body as a protective response to tissue damage, performing a local action at the site of release [24]. It has been attributed to anti-inflammatory, analgesic, anticonvulsant, antimicrobial, antipyretic, antiepileptic, immunomodulatory and neuroprotective properties [25,26,27,28], bringing therapeutic benefits in many disorders, such as chronic pain, neurodegeneration and inflammation [29,30]. In the dental field, PEA has also attracted considerable interest because of its remarkable properties; particularly, PEA could play a role in the management of the pain of orofacial origin, including BMS, OLP, periodontal disease, tongue a la carte and temporomandibular disorders (TMDs), as well as in the treatment of postoperative pain [31,32,33,34]. The addition of PEA-containing nutraceutical agents to scaling and root planning (SRP) in the treatment of periodontal disease appears to provide improvements in clinical and inflammatory parameters as well as post-treatment pain compared with SRP alone [32]. The use of exogenous PEA for the treatment of OLP needs further investigation for adequate treatment, as a reduction in inflammation in the disease by its local or systemic use has emerged [35]. In BMS, PEA provided significant benefits in terms of a significant reduction in burning sensation and intensity by treated patients [36] as well as in pain induced by osteoarthritis or TMJ arthralgia compared with ibuprofen [37]. 

Therefore, the main objective of the present study is to provide an overview of orofacial pain in its many manifestations and an updated analysis of the molecular pain-relieving and anti-inflammatory properties of PEA to understand its beneficial effects in the management of patients with orofacial pain, both neuropathic and nociceptive in nature. The aim is also to direct research toward the testing and use of other natural agents that have already been shown to have anti-inflammatory, antioxidant and pain-relieving actions and could offer important support in the treatment of orofacial pain. 

## 2. Materials and Methods

The articles included in this critical review were identified using the major search engines: Pubmed, Scopus and Google Scholar. The keywords used in all search engines were as follows: Oral facial pain and Palmitoylethanolamide; Oral facial pain and Natural agent. From the search for “Oral facial pain and Palmitoylethanolamide”, 4 articles were found on Pubmed, 648 results on Google Scholar and 62,153 on Scopus, covering the following time frame: 2000–2022. From the search for “Oral facial pain and Natural agent”, 54 articles were found on Pubmed, 17,900 results on Google Scholar and 37,238 on Scopus, covering the following time frame: 2000–2022. Of these articles, 97 were included in the following critical review after exclusion of duplicate papers and papers that did not fit the criteria for selection of papers. Titles and abstracts for inclusion were reviewed by at least two independent researchers. Full articles were requested for all articles that passed the initial screening. Each full article was evaluated by two researchers for final inclusion/exclusion. In case of disagreement, a third researcher was consulted, and the decision was made by consensus. Initial screening was based on the following criteria: RCTs, cohort studies, case-control studies and case series that included at least a sample number of 15, meta-analyses and systematic reviews. No studies were excluded based on language of publication.

## 3. Orofacial Pain

Pain represents a warning symptom against tissue damage or wound healing, which can be distinguished into primary or localized pain when it is felt in the area where the tissue damage occurred, or secondary or diffuse pain when it is felt in a different area from that where the tissue damage occurred [1,2]. In these cases, we speak of “healthy pain”, nociceptive and inflammatory pain of a traumatic, infectious or post-surgical nature [3]. Sometimes, however, pain may not be associated with a true biological benefit, as in chronic pain [38,39], which is defined as “unhealthy” and non-protective pain, including neuropathic or neurovascular and dysfunctional or centralized pain [3].

The term facial pain or orofacial pain refers to any type of pain in the area between the eyes and the jaw, including the oral cavity. Currently available data indicate a prevalence in the population of around 1.9%, with greater involvement of the female sex [4]. In 2020, the International Classification of Orofacial Pain (ICOP), which is the first hierarchical classification that uniquely addresses orofacial pain, was established and modeled on the International Classification of Headache Disorders (ICHD) (third edition, ICHD-3). It distinguishes six major classes of orofacial pain: pain in the dentoalveolar tissues and anatomically related, muscle pain, temporomandibular joint (TMJ) pain, neuropathic pain affecting cranial nerves, primary headache-like pain and idiopathic pain (Table 1) [5].

The evaluation, diagnosis and management of orofacial pain disorders is often a complex, multifactorial and multidisciplinary process involving several specialists. A detailed pain history, combined with a careful objective examination, is essential to properly treat facial pain and distinguish its origin.

## 4. Etiopathogenesis of Orofacial Pain

It is well known that pain perception represents a complex process involving bidirectional communication between the central and peripheral nervous systems. The nerve impulse originates as a result of the stimulation of the orofacial structures and is conducted along the pain afferent pathways of the cranial nerves: trigeminal (V) with its three branches ophthalmic (V1), maxillary (V2) and mandibular (V3); facial (VII); glossopharyngeal (IX); vagus (X); and by the cervical nerves (C1, C2 and C3). The trigeminal nerve is the most important nerve in the orofacial region. It is a highly sensory and reflexogenic nerve, which can easily be exposed to pathogenic noxa [5]. Based on etiopathogenesis, pain can be classified into nociceptive pain and neuropathic pain [8]. 

### 4.1. Nociceptive Orofacial Pain

The pain of a nociceptive nature is the most common in the orofacial region [8]. It is induced by the acute stimulation of nociceptors present in various regions of the body, including the oral cavity (oral mucosa, pulpal tissue and periodontal tissue), either directly, by mechanical, thermal or chemical stimuli, or by serving the mediators of inflammation. This includes algic syndromes of rheumatic, post-traumatic or post-surgical origin, which respond positively to therapy with anti-inflammatory drugs. Inflammatory pain is also referred to as nociceptive pain, as inflammation in the periphery sensitizes nociceptors and increases their spontaneous discharge frequency and their excitability to stimulation. Therefore, pain, clinically, cannot be considered only a system triggered by disease but rather a reflection of the overall excitability of nociceptive circuits [6]. The sensitivity of those nociceptive circuits can be affected by stimuli of various natures, and the state of excitability can determine the level of pain experienced [7,40]. It is known that tooth pain is, primarily, nociceptive and odontogenic in origin [7]. Odontogenic pain can originate from pulpal tissue, periodontal tissue (gingiva or periodontal ligament or alveolar bone) or oral mucosa [3]. Orofacial nociceptive pain could also originate from the structures of the temporomandibular joint (TMJ) [8]. 

#### 4.1.1. Toothache

Dental disease is the most frequent cause of acute facial pain. It is conveyed by the mandibular and maxillary branches of the trigeminal nerve and is characterized by deep, localized and detectable somatic pain. There are multiple diseases that induce orofacial pain of odontogenic origin [8]. Dentinal hypersensitivity is pain induced by exposure to dentin, following abrasion of enamel, or cementum, consequent to gingival recession or periodontal disease, causing resorption of alveolar bone. Pain is caused by stimuli of various kinds: thermal (hot or cold), tactile (brush or food contact), osmotic (sugary or salty foods) or dehydration (blowing air). Such stimuli cause movement of fluid in the dentinal tubules, which, by stimulating pulpal receptors, cause sharp, instantaneous pain that ceases upon termination of the stimulus. The prevalence in the adult population is 1:7, with a peak in young adults and a subsequent decrease with age [9]. Traumatic lesions to the dental elements can affect either the hard tissues, dental fractures with and without pulpal exposure or the supporting tissues of the dental elements, causing concussion, subluxation or dislocation of the teeth [41]. 

Pulpitis is the most frequent cause of trigeminal pain secondary to organic cause [9]. Pulpal inflammation, or pulpitis, produces increasingly intense and prolonged painful responses to thermal or osmotic stimulation, in contrast to the less painful and phasic responses observed with normal dentin sensitivity. Inflammatory responses at the initial stage may be reversible, but as the disease progresses, the process becomes irreversible and may lead to the development of spontaneous pain that occurs without provocation, probably due to both peripheral and central sensitization [42]. Odontogenic abscess with periapical (acute apical periodontitis) or periodontal (acute lateral periodontitis) origin is also a frequent cause of orofacial pain [43]. 

Alveolitis is osteitis that affects the dental socket after extraction, generally inducing the intense and continuous pain that occurs 2–4 days after avulsion. Pericoronitis is an infection of the pericoronary sac of a partially erupted tooth, frequently affecting the lower third molars [20]. Included or dystopic teeth, root fragments or foreign bodies, sclerotic bony areas and post-surgical pain are other causes that can cause symptomatic neuralgia [41]. 

#### 4.1.2. Lesions to the Oral Mucosa

Ulcerative and vesiculobullous stomatitis, oral lichen planus (OLP), traumatic lesions and other diseases affecting the oral mucosa are the cause of protopathic orofacial pain. Oral lichen planus (OLP) is a very common chronic inflammatory disease of the oral mucosa. The etiology of OLP is still uncertain; however, the most accepted hypothesis currently correlates it with an immunological process triggered by an antigen that alters the keratinocytes of the basal layer of the oral mucosa, making them susceptible to an immune cell response [10]. The latter results in the activation of T-lymphocytes, $\mathrm{CD}4^{+}$ and $\mathrm{CD}8^{+}$; although the first-line treatment option for OLP is topical (or sometimes systemic) steroids, their efficacy is variable, and there is a clear need for new treatment strategies [43]. The production of cytokines, such as interleukin-2 (IL-2), IL-4, IL-6, IL-8, interferon γ (INF-γ) and tumor necrosis factor α (TNFα), leads to apoptosis of keratinocytes [42]. Recently, it has been observed that in tissues with OLP, there is increased expression of cyclooxygenase-2 (COX-2) and, consequently, of all prostaglandins (PGs), and inflammatory mediators (TNFα and mast cells) induced by it [43,44]. Chankong et al. reported that immunohistochemical staining of COX-2 was approximately 1.4-fold higher in 25 patients with OLP than the corresponding staining in 13 control samples [45]. This finding would suggest how the overexpression of COX-2, which is responsible for PG production, may be related to the proposed autoimmune character of the disease [44,45,46,47,48].

#### 4.1.3. Temporomandibular Dysfunction (TMD)

Orofacial nociceptive pain could also originate from the temporomandibular joint (TMJ) structures. TMD pain may have different etiologies: degradation of TMJ structures, joint inflammation (i.e., degenerative joint disease—arthritis) and myofascial pain [11]. The latter, however, falls under central nervous system (CNS)-induced pain and not nociceptive pain, as mounting evidence negates a connection between peripheral inflammation in local muscles (e.g., temporalis, medial pterygoid and masseter) and myalgic pain. Diseases that induce nociceptive pain include malformative disorders due to growth alterations; synovitis and capsulitis consequent to extrinsic trauma, condylar movements forced by excessive tension, and extension by contiguous infection from neighboring tissues; retrodiscitis, i.e., inflammation of the retrodiscal tissue consequent to trauma or condylar–meniscal incoordination; traumatic, inflammatory and infectious osteoarthritis; and degenerative arthropathies (osteoarthritis) [11,44].

### 4.2. Neuropathic Orofacial Pain

Neuropathic pain is a chronic pain condition that may be attributable to a primary injury, a transient peripheral or CNS dysfunction, or disruption [13]. It can be classified as peripheral or central, depending on where it originates, and as episodic or continuous, depending on the duration of symptoms [45]. 

The true prevalence of neuropathic pain is not known. It has been estimated that 1–1.5% of the general population is affected [12].

Trigeminal neuralgia, atypical odontalgia, burning mouth syndrome (BMS), post-traumatic neuropathy, postherpetic neuralgia and complex regional pain syndrome are neuropathic pain conditions of the orofacial region. They are complex conditions to diagnose because, often, they are difficult to distinguish from dental disease, leading to unnecessary and resolving treatments such as endodontic treatment and the extraction of the tooth or teeth in the affected region [14]. Neuropathic pain responds to therapy with neurological drugs (anticonvulsants, antidepressants and neuroleptics), while anti-inflammatory drugs are of partial benefit [46].

#### 4.2.1. Trigeminal Neuralgia

Idiopathic trigeminal neuralgia is a facial algic disorder, unilateral in most cases (bilateral in 3–5% of cases), causing stabbing, short-lasting pain and reflex spasms of the facial muscles (tic douloureux). It mainly affects the second and third trigeminal branches, rarely the first (5%). Currently, it has been suggested that the organic cause of the condition is the compression of the trigeminal root by aberrant or tortuous vessels, i.e., a neurovascular conflict [47]. The diagnostic criteria used are those proposed by the IHS: paroxysmal attacks of facial or frontal pain lasting from a few seconds to up to 2 min. The pain has definite characteristics: distribution along one or more branches of the trigeminal; sudden, intense, superficial and aching, induced by the stimulation of trigger zones or by certain daily activities such as eating, talking or brushing the face or teeth; the patient is completely asymptomatic in the intermediate periods; and there is an absence of neurological deficits. Symptomatic trigeminal neuralgia is secondary to demonstrable structural disease, resulting in direct nerve distress in its intracranial (posterior cranial fossa) or extracranial course, such as angiomas, aneurysms, acoustic neurinomas, cholesteatomas, osteomas, adhesions, multiple sclerosis, brainstem infarcts and tumors. In these cases, the pain is topographically less precise and delimited; sensory neurological deficits may accompany it. The algic crisis is subcontinuous, with episodes of accentuation of varying duration (from 15 min to several hours) and, more rarely, hyperalgic accesses of short duration [48].

#### 4.2.2. Glossopharyngeal Neuralgia

Glossopharyngeal neuralgia is a rare condition that has features of onset and duration of pain common to those of trigeminal neuralgia. It differs from the latter in its localization: it affects the posterior third of the tongue, the tonsillar pillars, the entire pharynx, the Eustachian tube, the middle ear and behind the angle of the mandible. Swallowing and coughing represent the stimuli that most frequently trigger the attack. A distinction is also made for this one between idiopathic and symptomatic forms. In the latter form, pain persists during intercritical periods and may be accompanied by sensory deficits [49,50].

#### 4.2.3. Postherpetic Neuralgia, Post-Traumatic Neuropathies and Neuropathies of Neoplastic Origin

Pain induced by such diseases is also called deafferentation pain. It has the characteristic of occurring in the absence of nociceptive stimulus since it is generated by autonomic hyperexcitability of central sensory neurons in relation to the loss of peripheral afferents due to a nerve stem lesion. It is a typical pain of painful anesthesia, but also of neoplastic origin. Several lesions concerning the trigeminal nerve (with greater frequency affecting the inferior alveolar nerve) can be ascribed to this subgroup: invasion of the nerve trunk by inflammatory and infectious (osteomyelitis and herpes zoster) or neoplastic processes; compression or distortion by intracranial or extracranial diseases (tumors, aneurysms and vessels); and trauma (iatrogenic damage by intraneural section or injection and mandibular fractures) [51,52]. The diagnostic criteria, proposed by the International Headache Society (HIS), are as follows: pain is persistent in the distribution territory of one or more cranial nerves; demonstration of congruous injury; onset of pain in temporal relation to cranial nerve injury; and pain improves or disappears after spontaneous remission or after effective treatment of the injury [51,52].

#### 4.2.4. Atypical Odontalgia (AO)

Atypical odontalgia (AO), also called phantom tooth pain or non-odontogenic pain, is a disease characterized by continuous pain affecting the teeth or tooth orbits after extraction in the absence of any identifiable cause on a clinical basis or radiographic physical examination [53]. AO is a specific problem in the dental field and appears to be surprisingly complex [54].

#### 4.2.5. Burning Mouth Syndrome (BMS)

Burning mouth syndrome (BMS) is a syndrome characterized by burning and persistent oral pain, ranging from mild to intense, in the absence of organic disorders of the oral cavity and with a major impact on the quality of life of the sufferer [15]. It can be distinguished into primary, when no cause can be identified (idiopathic), and secondary, when it is related to an underlying medical syndrome, such as drug-related xerostomia, oral candidiasis, OLP, gastroesophageal reflux disease (GERD), migrating glossitis, smoking and intake of spicy or acidic foods or carbonated beverages. The differential diagnosis excludes other local and systemic factors that may induce burning pain within the oral mucosa [16]. Scala et al. proposed a set of differential diagnostic criteria to identify BMS. In this context, they established a distinction between basic and additional criteria for diagnosing the disease (Table 1) [55]. Although several etiologic theories have been proposed to explain BMS, none have received universal acceptance to date, and its origin remains unclear. Currently, several pieces of scientific evidence would suggest that underlying BMS would be alterations in some neuropathic mechanisms [56]. In detail, the presence of sensory alterations in patients with BMS indicates the existence of a biological basis related to alterations in both the central and peripheral nervous systems [57]. Blink reflex (BR) and thermal quantitative sensory testing (QST) have been used to evaluate the possible neuropathic involvement of patients with BMS in several studies [17,58]. The results showed that:

1. Higher BR stimulation thresholds were observed in patients with BMS, indicating dysfunction affecting tactile sensory fibers outside intraoral sites [18]. In addition, BR abnormalities were found in 20% of patients, suggesting possible subclinical brainstem disease and peripheral trigeminal neuropathy of the lingual or mandibular nerves [17].
2. QST abnormalities, understood as hypoesthesias, were observed in patients with BMS when the results obtained were compared with laboratory reference values [17,18,59]. These findings would indicate peripheral small-fiber neuropathy or deafferentation of the central trigeminal thermal pathways. Evidence supporting focal small-fiber neuropathy in BMS comes from neuropathologic studies showing decreased epithelial nerve fiber densities (ENFDs) of the mucosa of the tongue [58,60,61]. However, since thermal QST and ENFD are age-dependent, further studies taking this into consideration would be needed.

Recently, evidence of central nervous system involvement in BMS has emerged. BR alterations are attributable to impaired dopaminergic control, which is deficient in 20−36% of patients with BMS [17,18]. These alterations are confirmed by PET studies showing low levels of striatal dopamine in BMS [62]. This finding may partly explain pain perception in BMS, as several pieces of evidence support that the dopaminergic system and striatal dopamine D2 receptors (DRD2s) play an important role in central pain perception and in its modulation [62,63].

Further evidence supporting the neuropathic nature of BMS resides in several immunohistochemical studies, which have demonstrated the presence of both alterations in small-diameter (C) and larger-diameter nerve fibers, suggesting BMS could be a consequence of both generalized alterations and disturbances at different levels of the trigeminal system [64]. Indeed, patients diagnosed with BMS present with symptoms that are characteristic of trigeminal nerve disorders (alterations in pain perception and neuron transmission, and increased excitability of the trigeminal vascular system) [65]. 

However, although the neuropathic basis of the disorder is now commonly accepted, the influences of other factors such as:

- Psychological disorders (depression and anxiety) play an important role in pain modulation and perception, as they affect nerve transmission of pain and decrease the individual perception of pain [2]. A considerable percentage of BMS patients report depression and anxiety, and improvements in perceived symptomatology have been observed as a result of cognitive behavioral therapy and the use of anxiolytics [55]. This would indicate that psychological disorders might predispose to the development of BMS, although how they might influence its etiology remains unclear [2].
- Hormonal alterations associated with reduced estrogen and progesterone levels could promote mucosal dryness and the onset of psychological disorders. This is suggested by the observation that BMS has a higher prevalence in the female sex: middle-aged and postmenopausal women [66]. Supporting this hypothesis would be the good results in terms of xerostomia following oral hormone replacement therapy [67].

Most likely, BMS cannot always be traced back to the same cause. This observation is based on the different therapeutic responses shown by patients included in the different studies conducted to evaluate pharmacological substances for the treatment of BMS. In fact, if all cases of BMS were of the same origin, the drugs evaluated should have similar efficacy rates in all treated patients [65]. Nowadays, there are no universally accepted guidelines for the treatment of BMS, making it very difficult for healthcare professionals to manage affected patients [68].

### 4.3. Another Type of Pain: Sympathetic Reflex Pain 

Sympathetic reflex pain has the characteristic of self-maintaining through a vicious cycle involving the production of algogenic mediators by the sympathetic nervous system, which is activated by nociceptive afferents reaching the spinal cord via somatic sensory nerves; an example is myofascial pain [69]. It is a chronic pain condition characterized by the presence of the myofascial trigger point, a hyper-irritable pain point involving a limited number of muscle fibers. The literature suggests that myofascial trigger points should be considered generators of peripheral pain [70].

## 5. Palmitoylethanolamide

Palmitoylethanolamide (PEA) is a bioactive lipid mediator like endocannabinoids (eCBs) [22,23], which is included in the N-acyl-ethanolamine (NAE) family of fatty acid amides [71]. It is synthesized in all tissues of the body (including the encephalon) [24], within the lipid bilayer [72], as a pro-homeostatic protective response to tissue damage, performing a local action at the site of release [24]. It has been observed that PEA is usually found to be upregulated in many disease states [24]. Its pleiotropic effects currently include anti-inflammatory, analgesic, anticonvulsant, antimicrobial, antipyretic, antiepileptic, immunomodulatory and neuroprotective activities [25,26,27,28]. The multiple therapeutic effects of PEA result in the influence that its unique mechanisms of action exert on multiple pathways at different sites [28,72].

It has been observed how part of the anti-inflammatory activity of PEA can be attributed to its activity on the PPAR-α receptor. It belongs to the family of peroxisome proliferator-activated receptor transcription factors in the nuclear receptor superfamily, whose activation causes altered transcription of a large number of genes ranging from those encoding for proteins involved in fatty acid transport and metabolism to those encoding for pro-inflammatory molecules and oxidative stress [73,74]. PEA, like arachidonic acid or fibrate family compounds, is an agonist of PPAR-α, which promotes the repression of pro-inflammatory transcription factors such as NFκB, leading to the inhibition of the release of inflammatory cytokines, including tumor necrosis factor α (TNF-α) and interleukins 1β and 6 [73]. 

An additional target of PEA is the hydrolytic enzyme fatty acid amide hydrolase or FAAH. When PEA is present in high concentrations it competes with endogenous NAEs to bind to FAAH binding sites, thus preventing the hydrolysis of endogenous NAEs and increasing their levels [75]. Therefore, the anti-inflammatory activity of PEA would be attributable to the reduced degradation of endogenous NAEs rather than the inhibition of FAAH. This is supported by several studies showing that FAAH inhibitors have failed in clinical trials as analgesics [76]. Several studies have evaluated whether the beneficial effects of PEA are mediated or, alternatively, mitigated by its hydrolysis product, palmitic acid. It exhibits several biological activities, including the ability to affect Toll-like receptor signaling involved in macrophage activation in response to the presence of lipopolysaccharide [77] and that of inhibiting PPAR-α trans-activation although with a lower potency than PEA [78]. However, in an in vitro study, it was observed that blocking the hydrolysis of PEA did not reduce its action on prostaglandin production [79].

**Figure 1:**
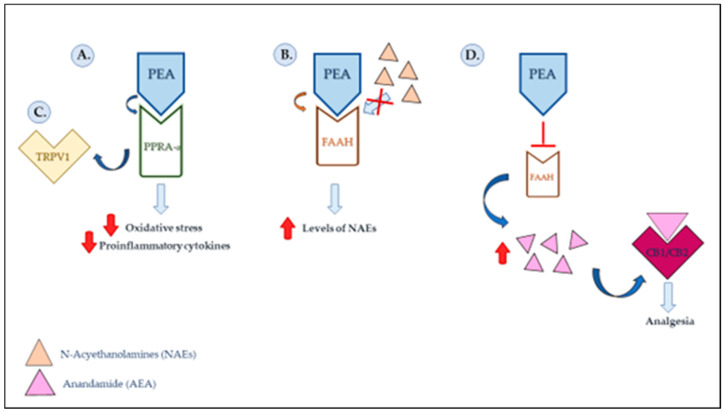
PEA’s mechanisms of action proposed for anti-inflammation and analgesia. (**A**) PEA is an agonist of the nuclear receptor PPAR-α, and its binding to it results in the repression of proinflammatory transcription factors, inducing a reduction in the release of inflammatory cytokines. (**B**) At high concentrations, PEA binds to the hydrolytic enzyme FAAH, competing with endogenous NAEs and reducing their degradation so as to promote the anti-inflammatory effect. (**C**) Through activation of PPAR-α, PEA can modulate the activity of a receptor channel (TRPV1), the dysfunction of which appears to be associated with several diseases, including neuropathic pain and inflammation. (**D**) PEA, by inhibiting FAAH expression, enables an increase in endogenous levels of the endocannabinoid, anandamide (AEA), presumably promoting analgesic action.

PEA activates and desensitizes vanilloid receptor 1 (TRPV1) channels [22]. It belongs to a family of receptor channels that determine transient changes in potential (TRPs or transient receptor potential channels) and are involved in the transduction of many chemical and physical stimuli, such as temperature, pressure, pH, smell, taste, vision and pain perception. It has been observed that TRP channel dysfunction is involved in several diseases, including neuropathic pain and inflammation [28]. It has been observed that PEA can modulate TRPV1 activity through several mechanisms, including the activation of PPAR- α and potentially acting as an allosteric modulator. In addition, it indirectly activates cannabinoid receptors 1 and 2 (CB1 and CB2) through the inhibition of the expression of FAAH, preventing the degradation of the endocannabinoid, anandamide (AEA), a phenomenon known as the “entourage effect” [71]. The main function of the CB2 receptor appears to be the control of inflammatory and nociceptive responses [27,71] (Figure 1).

The tissue-protective and anti-inflammatory activities of PEA have been recognized for many decades; however, scientific and clinical interest in it only increased after Rita Levi-Montalcini and her group discovered that PEA acts as a natural modulator of hyperactive mast cells, counteracting the pro-inflammatory actions of NGF [27]. In detail, it has been observed that lipid amides of the N-acylethanolamine type (such as PEA) are potential prototypes of naturally occurring molecules capable of modulating mast cell activation in vivo and exerting a local “anti-lesion” action, termed ALIA (autacoid local injury antagonism). Autacoid is a term used to refer to a regulatory molecule that is locally produced and controls metabolism locally [80]. PEA is an ALIAmide, an autocoid formed locally when inflammation or neurogenic pain occurs. The increase in PEA concentrations is based on the body’s own mechanisms for coping with pain and inflammation. The mast cell, soon after Levi-Montalcini’s discovery, proved to be an important target for the anti-inflammatory activity of PEA [81,82].

PEA’s multiple mechanisms of action generate therapeutic benefits in many disorders, including chronic pain neurodegeneration and inflammation [29,30]. Endogenous PEA is generally insufficient to counteract the chronic allostatic burden as observed in chronic inflammatory disorders, making exogenous administration a viable therapeutic strategy to increase endogenous levels and restore body homeostasis [29,83]. We currently find several products containing PEA commercially authorized for use as nutraceuticals (e.g., Neuridase^®^, Enfarma, Italy; PEA^®^, Longlife, Italy; and FIBROALGIL^®^, Herboplanet, Italy), dietary supplements or foods for medical purposes in several countries, with a generally recommended dose of 1200 mg/day [71].

### Palmitoylethanolamide in the Management of Diseases of Dental Interest

Several studies have explored the activity of PEA in various oral disorders, three including BMS, OLP, periodontal disease, map tongue and TMD [28,29,30]. The role of palmitoylethanolamide in the pathogenesis of various inflammatory diseases has also attracted considerable interest in the dental field. A promising role has also been observed in the treatment of postoperative pain [34].

The observation that periodontal ligament (PDL) cells induce local immune responses like microglia cells, which are involved in host defense mechanisms in neuroinflammatory events in the central nervous system, indicates that the immunological characteristics of PDL cells could be modulated by the endocannabinoid system, as seen for microglia [31]. In fact, it has been observed how adding a PEA-containing nutraceutical agent to scaling and root planing (SRP) in the treatment of periodontal disease improved clinical, inflammatory and post-treatment pain parameters compared with SRP alone [32].

It is known that PEA treatment, both in vivo and in vitro, can modulate COX-2 and PGs derived from it [84] and that COX-2 and PG levels are increased in OLP [35]. A recent study, which investigated the NAE system in relation to OLP, found that PEA synthesis is insufficiently mobilized in OLP to counterbalance the increased expression of COX-2 [84]. This insufficient mobilization of an endogenous, PEA-induced response to dampen inflammation in OLP opens the possibility that exogenous PEA could be a potentially useful treatment strategy for this disorder, given its anti-inflammatory properties (not least its ability to downmodulate mast cells to reduce inflammatory cytokine levels secondary to its effects on PPAR-α). Therefore, a clinical trial of appropriate local or systemic PEA treatment would greatly impact OLP [35].

Only recently, it has been observed that in individuals with BMS, there is a significant increase in TRPV expression and a decrease in neuropathic fibers, indicating that the condition represents a neuropathic process [60,85]. Plasma levels of PEA have been shown to be significantly higher in patients with BMS, compared with healthy individuals, and correlated with depressive symptomatology [86]. Several pieces of evidence support how PEA plays the action of an endogenous protective mediator, produced on demand by the body, to counteract inflammatory pain and neuronal cell damage in degenerative neuroinflammatory conditions [87]. In a model of chronic neuropathic pain induced in mice by chronic constriction injury of the sciatic nerve [88], PEA relieved neuropathic pain by acting on nociceptive receptors through modulation of non-neuronal cells, particularly mast cells and microglia, which contribute to the development and maintenance of chronic pain states by releasing algogenic mediators that interact with neurons to alter pain sensitivity [89]. 

The pain-relieving effect of ultramicronized PEA (um-PEA) has been further demonstrated in a cohort of more than 600 patients with chronic pain due to various conditions, including nerve root compression, osteoarthritis, post-herpetic neuralgia, diabetic polyneuropathy and pain, associated with chemotherapy-induced neuropathy. However, only recently, a study investigated PEA’s role in the management of BMS patients, noting that treated patients reported a significant decrease in reported burning sensation and intensity. These findings, however, should be further investigated, as this is only a preliminary study with a very limited number of patients [36].

In a randomized clinical trial comparing the effect of PEA versus ibuprofen (NSAIDs) in reducing pain induced by osteoarthritis or arthralgia of the temporomandibular joint (TMJ), PEA was effective in the anti-inflammatory treatment of TMJ. After 2 weeks of treatment, pain reduction, along with an improvement in maximum mouth opening, was significantly greater in the PEA-treated group than in the ibuprofen-treated group [37]. 

We currently find several nutraceutical compounds on the market in which PEA is found ultramicronized (to increase its bioavailability) and in combination with other substances with pharmacological activity. In a recent study conducted on rats, the activity of a nutraceutical agent containing PEA and acetyl-l-carnitine (LAC) was evaluated [90]. The results showed that they exerted greater anti-nociceptive and anti-inflammatory effects, inhibiting the production of associated inflammatory mediators compared with the administration of LAC alone than with the separate administration of LAC and PEA. In another study conducted on rats, the combination of PEA and polydatin (PLD), a natural precursor of resveratrol, was tested and has antioxidant activity [91]. The results showed that this association showed greater anti-inflammatory and anti-hyperalgesic effects than PEA alone. Finally, in a study conducted on periodontal patients, the activity of a nutraceutical agent containing PEA, Bromelain and Baicalin was evaluated. The results showed that the combination of SRP and the nutraceutical agent induced a greater reduction in periodontal parameters than SRP alone, demonstrating the important anti-inflammatory activity of the nutraceutical agent (Table 2) [32]. 

## 6. Other Natural Agents with Potential in the Treatment of Pain 

Over the past few years, multiple natural agents have been considered for their therapeutic properties. The genus *Ficus* is one of the largest genera in the mulberry family (Moraceae) and appears to be rich in flavonoids, triterpenoids, phenols, coumarins, anthocyanins and glycosides that are responsible for their antimicrobial, nematicidal, antioxidant, antidiabetic, antidiarrheal, anthelmintic, antitumor, anti-inflammatory, antiplasmodial, antiulcer, antipyretic and gastroprotective activities [92]. *Kebergia* (Meliaceae) is a genus of flowering shrubs and trees widely distributed on the African continent, which contains metabolites, such as limonoids, triterpenoids, coumarins, steroids, alkaloids, stilbenes and phenolic compounds, with marked pharmacological activities, including antiplasmodial, antimicrobial, antiproliferative and uterotonic activities [93]. The species of the genus Eremostachys have shown important anti-free radical, antioxidant and antibacterial effects [94]. Perovskia is a small genus in the Lamiaceae family that includes a variety of promising medicinal properties, including antimicrobial and antioxidant properties [95]. *Aesculus hippocastanum* and *Scutellaria baicalensis* are two natural agents that have active ingredients frequently found in association with PEA. In the next two subsections, there is a description of these natural compound agents.

### 6.1. Compounds Derived from Aesculus Hippocastanum

Horse chestnut (*Aesculus hippocastanum*) is a tree from whose seeds, bark and leaves various extracts and phytoconstituents can be derived. The most represented are the flavonoids quercetin, kaempferol and escin [96].

Flavonoids represent a large class of phenolic compounds widespread in the plant world. Chemically, most flavonoids consist of a central skeleton composed of 15 carbon atoms (C-15) and, more specifically, 3 rings: 2 benzyl rings, named A and B, and 1 heterocyclic ring, named C [97]. Flavonoid compounds have been shown to have numerous biological effects, including neuroprotective, antioxidant, anti-inflammatory, antimicrobial and cardioprotective effects [98]. In recent years, particular attention has been focused on these compounds’ effects on various types of neuropathies and neuropathic pain [99]. Among all the 6000 flavonoid compounds known today, many have been shown to be effective in ameliorating neuropathic pain through different mechanisms, for example, by the down-regulation of cytokines, including TNF-α, or through interactions with the GABAergic and dopaminergic systems [98].

Escin is the main bioactive compound derived from horse chestnut; chemically, it is acid triterpene glycoside and possesses anti-inflammatory, antiedematous and vessel tonic effects [100]. It can increase the resistance and decrease the permeability of capillaries; because of this characteristic, it is used in the treatment of various types of edema, varicose veins and hemorrhoids; hematomas and bruises; and capillary fragility. In addition to the traditional uses, the literature currently shows numerous other pharmacological effects of horse chestnut extracts [96]. These include positive effects on obesity, diabetic neuropathy, gastro- and hepatoprotective action, antimicrobial action, anti-inflammatory action and even positive effects on periodontitis [96,101]. Research has already shown, in part, how these compounds, especially escin, are able to exert all these actions, but many mechanisms are still not fully understood, and therefore, it will still take efforts to understand how they can be used in the treatment of different diseases.

### 6.2. Compounds Derived from Scutellaria Baicalensis 

*Scutellaria baicalensis* is a plant from whose roots an extract is obtained that is particularly rich in flavonoids, the major ones being baicalin and its aglycone, baicalein. Like many other compounds belonging to the flavonoid family, both have been shown to have several pharmacological effects [102]. The most important are antioxidant and anti-inflammatory effects, on which others often depend, such as anti-cancer, anti-diabetes, anti-thrombotic, anti-cardiovascular, hepatoprotective and neuroprotective effects [103].

Specifically, it has been shown that these compounds can be used to treat inflammatory disorders because they regulate the levels of many pro-inflammatory cytokines such as IL-1β, IL-18 and TNF-α [103,104]. They can also act directly on various immune cells, including lymphocytes, macrophages, monocytes and neutrophils, by promoting the production of anti-inflammatory cytokines. Countless are the molecular mechanisms by which they can act, including the inhibition of arachidonic acid metabolism, and the down-regulation of important molecular pathways (MAPK, Akt, NFκB and JAK-STAT), but there are still many aspects to be elucidated [104]. Possible positive effects on neurological and cerebrovascular disorders are also being investigated for this compound. Current findings lead to the conclusion that baicalin has a neuroprotective role that may be direct or indirect through its anti-inflammatory properties [105]. Certainly, we need to investigate what all the possible targets of these compounds may be, improve their bioavailability and pharmacokinetics and better understand any long-term toxic effects.

## 7. Conclusions

Nowadays, the role of natural agents in the treatment of many diseases is certainly a central theme in medical research. Every year, numerous studies explore the potential of different molecules with the aim of finding effective alternatives to traditional drugs, which are often accompanied by too many adverse effects. 

Palmitoylethanolamide is an example of a molecule of natural origin that has attracted the interest of the scientific community due to its involvement in numerous inflammatory mechanisms. Today, this endogenous factor can be supplemented through different formulations indicated most often for the treatment of chronic pain in different fields of medicine. Specifically, ultramicronized PEA has been found to be particularly effective against pain symptoms following osteoarthritis, post-herpetic neuralgia, diabetic polyneuropathy and pain associated with chemotherapy-induced neuropathy. Although there are already studies in the literature that have investigated the effect of PEA in various oral cavity disorders, there is still a lack of valid evidence on the possible therapeutic implications of this molecule in the dental field. Therefore, in the future, there is a need for the scientific community to conduct more research and trials that may lead to the use of PEA for orofacial pain management. Lately, research efforts are focused on understanding the pharmacological activities of numerous other natural agents, including many belonging to the flavonoid family. These are metabolites that can be obtained from any plant, fruit or vegetable. Many of these are brought to market often associated with each other in the form of dietary supplements and used in various areas of medicine, usually as adjuncts to traditional therapies. Famous, for example, are the compounds obtained from *Aesculus hippocastanum* and *Scutellaria baicalensis.* These natural compounds may also have potential benefits in orofacial neuropathic pain, although further studies are needed to specifically investigate their action in this regard. 

In conclusion, we believe that natural agents offer important support in the treatment of orofacial pain by virtue of their important anti-inflammatory, antioxidant and pain-relieving actions, which allow them to act on pathologies with different spectrums that are often accumulated by an underlying inflammatory state.

## Figures and Tables

**Table 1:** Classification of neuropathic pain according to the ICOP 2020 classification [20].

CLASSIFICATION	EXPLANATION
1. OROFACIAL PAIN ATTRIBUTED TO DISORDERS OF DENTOALVEOLAR AND RELATED ANATOMICAL STRUCTURES.	Pain caused by disease, injury or abnormal functioning of the tooth pulp, periodontium, gingiva, oral mucosa, salivary glands or jaw tissue, or pain resulting from the normal functioning of the tooth pulp that signals the risk of tooth damage. - Dental pain; - Pain in the oral mucosa, salivary glands and jaw bones.
2. MYOFASCIAL OROFACIAL PAIN.	Localized pain in the masticatory muscles, with or without functional impairment. - Primary orofacial myofascial pain; - Secondary orofacial myofascial pain.
3. TEMPOROMANDIBULAR JOINT (TMJ) PAIN.	Localized TMJ pain, occurs at rest or during movement or palpation of the jaw. - Primary pain of the temporomandibular joint; - Secondary pain of the temporomandibular joint.
4. OROFACIAL PAIN ATTRIBUTED TO CRANIAL NERVE INJURY OR DISEASE.	A localized pain in the distribution area of one of the cranial sensory nerves (i.e., the trigeminal and glossopharyngeal nerves) with a history of trauma or disease known to cause nerve injury. - Pain attributed to injury or disease of the trigeminal nerve; - Pain attributed to injury or disease of the glossopharyngeal nerve.
5. OROFACIAL PAIN SIMILAR TO PRIMARY HEADACHE PRESENTATIONS.	Pain in the orofacial area, similar to one of the primary headache types in character, duration, and intensity of pain with or without the symptoms associated with these headache types but without concomitant headache. - Orofacial migraine; - Orofacial pain of the tension type; - Trigeminal autonomic orofacial pain; - Neurovascular orofacial pain.
6. IDIOPATHIC OROFACIAL PAIN.	Unilateral or bilateral intra-oral or facial pain in the distribution of one or more branches of the trigeminal nerve for which the etiology is unknown. - Burning mouth syndrome (BMS); - Persistent idiopathic facial pain (PIFP); - Persistent idiopathic dentoalveolar pain; - Constant unilateral facial pain with additional attacks (CUFPA).

**Table 2:** Summary of PEA’s effects in the management of diseases of dental interest.

Oral Disorder	Outcomes	Refs	Year
Periodontal disease	Improvement in clinical inflammatory and post-treatment pain after SRP.	[32]	2021
OLP	Potential reduction in inflammation through the decrease in levels of pro-inflammatory cytokines.	[35]	2020
BMS	Neurological pain relief.	[89]	2013
Osteoarthritis or arthralgia of TMJ	Pain reduction and improvement in maximum mouth opening.	[37]	2012

## Data Availability

Data are available from the corresponding author upon reasonable request.
